# Toxic epidermal necrolysis and Stevens-Johnson syndrome

**DOI:** 10.1186/1750-1172-5-39

**Published:** 2010-12-16

**Authors:** Thomas Harr, Lars E French

**Affiliations:** 1 Department of Dermatology, University Hospital Zurich, Switzerland

## Abstract

Toxic epidermal necrolysis (TEN) and Stevens Johnson Syndrome (SJS) are severe adverse cutaneous drug reactions that predominantly involve the skin and mucous membranes. Both are rare, with TEN and SJS affecting approximately 1or 2/1,000,000 annually, and are considered medical emergencies as they are potentially fatal. They are characterized by mucocutaneous tenderness and typically hemorrhagic erosions, erythema and more or less severe epidermal detachment presenting as blisters and areas of denuded skin. Currently, TEN and SJS are considered to be two ends of a spectrum of severe epidermolytic adverse cutaneous drug reactions, differing only by their extent of skin detachment. Drugs are assumed or identified as the main cause of SJS/TEN in most cases, but Mycoplasma pneumoniae and Herpes simplex virus infections are well documented causes alongside rare cases in which the aetiology remains unknown. Several drugs are at "high" risk of inducing TEN/SJS including: Allopurinol, Trimethoprim-sulfamethoxazole and other sulfonamide-antibiotics, aminopenicillins, cephalosporins, quinolones, carbamazepine, phenytoin, phenobarbital and NSAID's of the oxicam-type. Genetic susceptibility to SJS and TEN is likely as exemplified by the strong association observed in Han Chinese between a genetic marker, the human leukocyte antigen HLA-B*1502, and SJS induced by carbamazepine. Diagnosis relies mainly on clinical signs together with the histological analysis of a skin biopsy showing typical full-thickness epidermal necrolysis due to extensive keratinocyte apoptosis. Differential diagnosis includes linear IgA dermatosis and paraneoplastic pemphigus, pemphigus vulgaris and bullous pemphigoid, acute generalized exanthematous pustulosis (AGEP), disseminated fixed bullous drug eruption and staphyloccocal scalded skin syndrome (SSSS). Due to the high risk of mortality, management of patients with SJS/TEN requires rapid diagnosis, evaluation of the prognosis using SCORTEN, identification and interruption of the culprit drug, specialized supportive care ideally in an intensive care unit, and consideration of immunomodulating agents such as high-dose intravenous immunoglobulin therapy. SJS and TEN are severe and life-threatening. The average reported mortality rate of SJS is 1-5%, and of TEN is 25-35%; it can be even higher in elderly patients and those with a large surface area of epidermal detachment. More than 50% of patients surviving TEN suffer from long-term sequelae of the disease.

## Background, disease name and synonyms

Stevens-Johnson syndrome (SJS) was first described in 1922, as an acute mucocutaneous syndrome in two young boys. The condition was characterized by severe purulent conjunctivitis, severe stomatitis with extensive mucosal necrosis, and purpuric macules. It became known as SJS and was recognized as a severe mucocutaneous disease with a prolonged course and potentially lethal outcome that is in most cases drug-induced, and should be distinguished from *erythema multiforme (EM) majus*. Recent clinical studies have shown that the term 'EM majus' should not be used to describe SJS as they are distinct disorders [1-4].

In 1956, Alan Lyell described four patients with an eruption resembling scalding of the skin which he called toxic epidermal necrolysis or TEN [4]. It was only as more patients with TEN were reported in the years following Lyell's original publication, that it became clear that TEN was drug induced, and that certain drugs such as sulfonamides, pyrazolones, barbiturates, and antiepileptics were the most frequent triggers of TEN. Increasingly to date, SJS and TEN are considered to be two ends of a spectrum of severe epidermolytic adverse cutaneous drug reactions, differing only by their extent of skin detachment.

## Epidemiology

SJS and TEN are rare diseases in absolute numbers with an incidence of 1.89 cases of TEN per million inhabitants per year reported for Western Germany and Berlin in 1996 [5]. La Grenade et al report similar results, with 1.9 cases of TEN per million inhabitants per year based on all cases reported to the FDA AERS database in the USA [6]. Lower incidence rates were reported by Chan et al in Singapore [7]. Certain infectious diseases may have an impact on the incidence of TEN, and this is clearly the case for HIV where the annual incidence is approximately 1000-fold higher than in the general population, with approximately 1 case per thousand per year in the HIV-positive population ([8]. In a study of HIV positive patients of the greater Paris area in the late eighties and early nineties, 15 cases of SJS/TEN were reported in patients with AIDS compared to 0.04 expected cases [9]. In another study only ten out of 50 cases of SJS/TEN in HIV patients could be clearly attributed to the use of medications, whereas in the other cases a cause could not be determined due to lack of data of drug intake or details [10].

Regional differences in drug prescription, the genetic background of patients (HLA, metabolizing enzymes), the coexistence of cancer, or concomitant radiotherapy [11,12], can have an impact on the incidence of SJS and TEN.

To a lesser extent, other infections have occasionally been reported as the sole cause. Mycoplasma pneumoniae infections are widely documented to cause SJS and TEN without initial exposure to drugs [13-15]. Furthermore, Herpes simplex virus was recognized in several cases of SJS, especially in children [16]. Single case reports describe Lupus erythematodes [17] or reactivation of Herpes simplex under treatment with azithromycine as potential causes of SJS [18]. The occurrence of TEN in a patient with severe aplastic anaemia after allogeneic haematopoietic stem cell transplantation has also been reported [19]. However there are still cases of SJS/TEN without any obvious identifiable cause.

## Clinical Features

### Acute Phase

Initial symptoms of toxic epidermal necrolysis (TEN) and Stevens Johnson Syndrome (SJS) can be unspecific and include symptoms such as fever, stinging eyes and discomfort upon swallowing. Typically, these symptoms precede cutaneous manifestations by a few days. Early sites of cutaneous involvement are the presternal region of the trunk and the face, but also the palms and soles. Involvement (erythema and erosions) of the buccal, genital and/or ocular mucosa occurs in more than 90% of patients, and in some cases the respiratory and gastrointestinal tracts are also affected [20,21]. Ocular involvement at the onset of disease is frequent, and can range from acute conjunctivitis, eyelid edema, erythema, crusts, and ocular discharge, to conjunctival membrane or pseduomembrane formation or corneal erosion, and, in severe cases, to cicatrizing lesions, symblepharon, fornix foreshortening, and corneal ulceration [22,23]. The severity of acute ocular manifestation is not however predictive of late complications [24]. The morphology of early skin lesions includes erythematous and livid macules, which may or may not be slightly infiltrated, and have a tendency to rapid coalescence (Table 1). The above mentioned skin signs associated with mucosal involvement are clear danger signs and warrant the initiation of rapid diagnostic confirmation with immediate cryosections of a skin biopsy. Histological examination including direct immunofluorescence analysis of the skin biopsy is also important in order to rule out differential diagnoses such as autoimmune blistering diseases, bullous fixed drug eruption, acute generalized exanthematic pustulosis, and due to its rarity in adults, to a lower extend staphyloccocal scalded skin syndrome.

**Table 1:** clinical features that distinguish sjs, sjs-ten overlap, and ten (adapted after 1)

Clinical entity	SJS	SJS-TEN overlap	TEN
Primary lesions	Dusky red lesions	Dusky red lesions	Poorly delineated erythematous plaques
	Flat atypical targets	Flat atypical targets	Epidermal detachment
			Dusky red lesions
			Flat atypical targets

Distribution	Isolated lesions	Isolated lesions	Isolated lesions (rare)
	Confluence (+) on face and trunk	Confluence (++) on face and trunk	Confluence (+++) on face, trunk, and elsewhere

Mucosal involvement	Yes	Yes	Yes

Systemic symptoms	Usually	Always	Always

Detachment (%body surface area)	< 10	10-30	> 30

In a second phase, large areas of epidermal detachment develop. In the absence of epidermal detachment, more detailed skin examination should be performed by exerting tangential mechanical pressure on several erythematous zones (Nikolsky sign). The Nikolsky sign is positive if mechanical pressure induces epidermal detachment, but is not specific for TEN or SJS, as it can also be positive in, for example, autoimmune bullous skin diseases.

**Figure 1:**
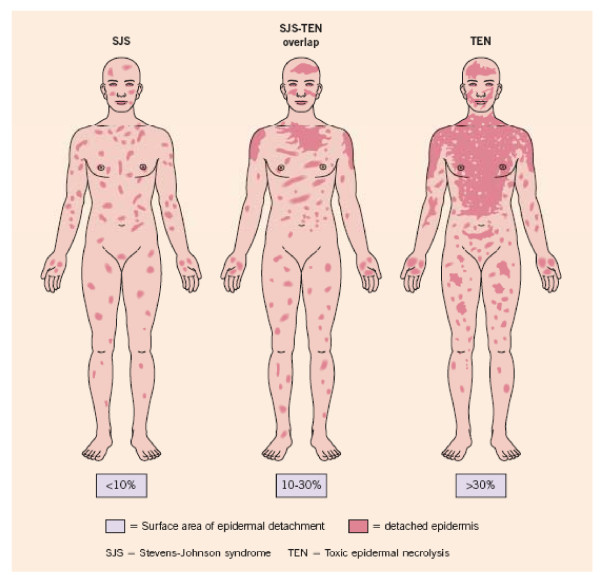
Pictural representation of SJS, SJS-TEN overlap and TEN showing the surface of epidermal detachment (Adapted from Fig 21.9 Bolognia and Bastuji-Garin S. et al. Arch Derm 129: 92, 1993)

The extent of skin involvement is a major prognostic factor. It should be emphasized that only necrotic skin, which is already detached (e.g. blisters, erosions) or detachable skin (Nikolsky positive) should be included in the evaluation of the extent of skin involvement. Bastuji-Garin et al. proposed classifying patients into three groups according to the degree of skin detachment (Table 1, Figure 1) [1].

### Late phase and sequelae

Sequelae are common features of late phase TEN. According to the study of Magina et al [25] following symptoms are found: hyper- and hypopigmentation of the skin (62.5%), nail dystrophies (37.5%), and ocular complications. According to a study of Yip et al. 50% of patients with TEN develop late ocular complications including, by order of decreasing frequency, severe dry eyes (46% of cases), trichiasis (16%), symblepharon (14%), distichiasis (14%), visual loss (5%), entropion (5%), ankyloblepharon (2%), lagophthalmos (2%), and corneal ulceration (2%) [24]. Hypertrophic scars are only seen in very few patients [26]. Long-term complications of mucosal involvement occur in 73% of patients who present mucosal involvement in the acute phase, and the mucosal sequelae involve mainly the oral and oesophageal mucosa, and to a lesser extent lung and genital mucosa [27]. In a small post SJS/TEN study seven out of nine patients had either xerostomia or keratoconjunctivitis or both, resembling Sjögren-like syndrome [28]. Additionally, another group reported a patient with Sjögren-like pluriglandular exocrine insufficiency including exocrine pancreatic impairment [29].

## Etiology and pathogenesis

### Genetic susceptibility

Genetic factors associated with drug hypersensitivity are a complex issue that has been studied in different populations and a variety of ethnic backgrounds. A unique and strong association between HLA, drug hypersensitivity and ethnic background was discovered by Chung et al. who showed a strong association in Han Chinese between the HLA-B*1502, SJS and carbamazepine [30]. This high association with an odds ratio of 2504 led to further studies in a similar ethnical group of Hong Kong Han Chinese with severe adverse reactions to antiepileptic drugs [31]. Another study confirmed the susceptibility of individuals with HLA-B*1502 to carbamazepine in a Thai population [32]. A smaller Indian based study however showed only a weak correlation between HLA-B*1502 and carbamazepine induced severe drug allergy. A genetic correlation could not however be shown in Japanese or Europeans [33-35]. Indeed, in a large European study (RegiSCAR), HLA-B genotyping was performed in patients with severe cutaneous adverse reactions caused by the two previously mentioned drugs (carbamazepine, allopurinol) and another three high risk drugs (sulfamethoxazole, lamotrigine, NSAID's of oxicam-type). This RegiSCAR study revealed that HLA-B*1502 is neither a marker for carbamazepine, sulfamethoxazole, lamotrigine, or NSAID's of oxicam-type induced SJS/TEN nor a sufficient explanation for the cause of the disease in Europeans [35,36]. This leads to the conclusion that this genetic constellation (HLA-B*1502) is not a population independent marker for SJS/TEN in carbamazepine exposed individuals. Severe cutaneous reactions in HLA-B*1502 subjects were not only associated with the drug carbamazepine, but also, to a lesser extent (lower odds ratio), with phenytoin and lamotrigine [31].

A second strong association between HLA genotype and SJS/TEN has been reported for allopurinol. Indeed, 100% of Han Chinese patients with a severe adverse drug reaction to allopurinol were HLA-B*5801 positive [37]. Subsequently, a strong association between SJS/TEN and HLA-B*5801 was found in Japanese patients [34], Thai patients [32], and also, to a lesser extent (55% of cases), in patients of European origin [36].

### Pathomechanism of SJS/TEN

The pathogenesis of SJS/TEN is not fully understood but is believed to be immune-mediated, as re-challenging an individual with the same drug can result in rapid recurrence of SJS/TEN [38,39]. The histopathology of SJS/TEN lesions show that keratinocyte apoptosis followed by necrosis is the pathogenic basis of the widespread epidermal detachment observed in SJS/TEN. The clinical, histopathological and immunological findings in SJS/TEN support the currently prevalent concept, that SJS and TEN are specific drug hypersensitivity reactions in which cytotoxic T lymphocytes (CTL) play a role in the initiation phase. Indeed, in the early phase of disease, blister fluid contains mainly cytotoxic CD8+T lymphocytes [40,41], suggesting that a major histocompatibility (MHC) class-I restricted drug presentation leads to clonal expansion of CD8+ CTLs, and the subsequent - to date only incompletely understood - immune reaction that causes SJS/TEN. These CD8+ T cells express common cutaneous leukocyte antigen (CLA) and are negative for CD45RA and CD28. Nassif et al. were able to demonstrate that blister T cells from patients exert drug specific cytototoxic activity against both autologous B-lymphocyte cell lines and keratinocytes [42], and furthermore demonstrated that this cell-mediated cytotoxicity was mediated by granzyme B. The discrepancy between the paucity of the infiltration of immune cells (including CTLs) in the skin of patients with SJS/TEN and the overwhelming keratinocyte apoptosis has however lead to the search for cytotoxic proteins and/or cytokines that may "amplify" the extent of keratinocyte apoptosis that CTLs alone could induce upon cell-cell contact. To date, the strongest evidence suggests a key contribution of the cytotoxic molecules FasL and granulysin as molecules responsible for the disseminated keratinocyte apoptosis in SJS/TEN [43,44].

The role of the membrane form of the death ligand FasL and its cognate death receptor Fas in the signalling that triggers keratinocyte apoptosis is supported by research performed using an ex-vivo experimental set up with TEN lesional skin biopsy cryostat section overlays with Fas-expressing lymphoid target cells [44]. However, the functional relevance of up-regulated keratinocyte membrane FasL, and thus its ability to induce keratinocyte cell death, has been questioned by some as the above ex-vivo demonstration of the lytic ability of keratinocyte FasL in TEN was limited in its effect on lymphoid target cells and not demonstrated with keratinocytes as target cells. It is well known that primary keratinocytes are sensitive to the cytolytic effect of FasL in vitro, and this sensitivity can be further enhanced by interferon gamma, a cytokine known to be present in the skin during TEN [45-47]. However, it is still not fully understood what causes the up-regulation of FasL/Fas on keratinocytes, and how the immune system, including T cells found in blister fluid at the onset of disease may regulate this.

The role of soluble FasL (sFasL) in SJS/TEN remains controversial. It appears clear now that increased levels of sFasL can be found in the serum of patients with SJS/TEN, and levels of sFasL are consistently elevated when analysis is performed preceding skin detachment [48]. Soluble FasL as opoposed to membrane-bound FasL is, however, very poorly cytolytic, and it is therefore unlikely to be a cause of keratinocyte apoptosis in TEN [49]. Nevertheless, one study showed that sera of SJS/TEN were able to induce abundant keratinocyte apoptosis and furthermore that peripheral blood monuclear cells of patients stimulated by the causative drug excreted high levels of sFasL [50], but it should be noted that sera can contain small membrane vesicles with membarne bound FasL that can account for the observed activity.

Gene expression analysis of blister fluid cells, and analysis of blister fluid from patients with SJS/TEN has also recently identified secretory granulysin (a cationic cytolytic protein secreted by CTL's, NK cells and NKT cells) as a key molecule responsible for the induction of keratinocyte death in TEN [43]. Blister fluid cells express high levels of granulysin mRNA, the protein is found in increased concentrations in blister fluid, and most importantly, recombinant granulysin mimicks features of SJS/TEN when injected intradermally in mice. The finding that elevated serum granulysin levels apparently discriminate between serious and non-blistering adverse drug reactions, serum granulysin levels being normal in the latter, lends further support an important role of granulysin in SJS/TEN [51].

In conclusion, and based on our knowledge to date, CD8 T-cells as well as the cytolytic molecules FasL and granulysin are key players in the pathogenesis of SJS/TEN. How a culprit drug in a given patient who will develop SJS/TEN regulates the function of these key players is the subject of ongoing research.

### Drugs

Drug exposure and a resulting hypersensitivity reaction is the cause of the very large majority of cases of SJS/TEN. In absolute case numbers, allopurinol is the most common cause of SJS/TEN in Europe and Israel [52], and mostly in patients receiving daily doses of at least 200 mg.

In a case control study of patients hospitalized for SJS/TEN in selected hospitals in France, Germany, Italy and Portugal between 1989 and 1993, Roujeau et al. reported that the following drugs are at increased risk of inducing SJS/TEN when used over a short period of time: trimetroprim-sulfamethoxazole and other sulfonamide-antibiotics, aminopenicillins, cephalosporins, quinolones and chlormezanone. Among drugs usually taken for longer periods of time (carbamazepine, phenytoin, phenobarbital, valproic acid, non-steroidal antinflammatory drugs of the oxicam-type, allopurinol and corticosteroids), the highest risk of induction of SJS/TEN occurs during the first 2 months of treatment with a sharp drop of incidence thereafter [8]. However, although these drugs have a high relative risk compared to other drugs, the actual risk remained low with 5 cases or less per million users per week. A similar population was studied between 1997 and 2001 by Mockenhaupt et al. in a multinational case-control study in Europe covering more than 100 million inhabitants in which special attention was given to newly marketed drugs [53]. This study identified nevirapine, lamotrigine, and sertraline, as drugs with a significantly increased risk of inducing SJS/TEN. Older drugs identified as having a high risk of inducing SJS/TEN were sulfamethoxazol/trimethoprim (SMX/TMP), sulfonamides (sulfasalazine, sulfadiazine, sulfadoxine, sulfafurazole), allopurinol, carbamazepine, phenytoin, phenobarbital, and NSAID's of the oxicam-type (meloxicam, piroxicam, tenoxicam). However the incidence of SJS/TEN under treatment with valproic acid is confounded by the concomitant use of other drugs, such as lamotrigine [5]. Mockenhaupt et al. were able to show that almost all cases of SJS/TEN developed within 63 days of starting use of antiepileptic drugs, and that the risk of developing SJS/TEN per 10 000 new users was significantly increased for carbamazepine (1.4 cases per 10 000 users), lamotrigine (2.5), phenobarbital (8.1) and phenytoine (8.3). The incidence for valproic acid was low compared to other antiepileptic drugs with 0.4 cases per 10 000 users [54]. Furthermore, studies in different populations indicate that the risk of developing SJS/TEN is highest when the drug has been recently initiated, and subsequently declines within 8 weeks or more of administration [5,55]. Interestingly the long term use of glucocorticosteroids for a variety of diseases does not change the incidence of the occurrence of SJS/TEN for the incriminated drugs, but it appears that glucocorticoids lengthen the interval between the beginning of the intake of the drug and onset of SJS/TEN [56]. A recent survey of TEN in children identified similar drugs to those in adults, as well as a possibly increased susceptibility to acetaminophen (paracetamol) [57].

Photo-induced TEN or SJS are only reported in very rare cases. Case reports exist for hydroxchloroquine [58], naproxene [59] and clobazam [60]. An often addressed issue is the induction of TEN or SJS after vaccination. The vaccine adverse event reporting system concludes that despite the plausibility of a relationship between vaccination and SJS/TEN, the very small number of reports compared to the large amount of vaccinations and the benefits of vaccinations outweighs the potential risk of SJS/TEN [61].

### Diagnosis and diagnostic methods

The diagnosis relies on the one hand on clinical symptoms and on the other hand on histological features. Typical clinical signs initially include areas of erythematous and livid macules on the skin, on which a positive Nikolsky sign can be induced by mechanical pressure on the skin, followed within minutes to hours by the onset of epidermal detachment characterized by the development of blisters. It should be noted, however, that the Nikolsky sign is not specific for SJS/TEN. Mucosal, including ocular, involvement develops shortly before or simultaneously with skin signs in almost all cases. To distinguish SJS, SJS-TEN and TEN the surface area of the detachment is the main discriminating factor (Figure 1). Histological work up of immediate cryosections or conventional formalin-fixed sections of the skin revealing wide spread necrotic epidermis involving all layers confirms the diagnosis. In order to rule out autoimmune blistering diseases, direct immune fluorescence staining should be additionally performed and no immunoglobulin and/or complement deposition in the epidermis and/or the epidermal-dermal zone should be detected.

### Differential diagnosis

Major differential diagnosis of SJS/TEN are autoimmune blistering diseases, including linear IgA dermatosis and paraneoplastic pemphigus but also pemphigus vulgaris and bullous pemphigoid, acute generalized exanthematous pustulosis (AGEP), disseminated fixed bullous drug eruption and staphyloccocal scalded skin syndrome (SSSS). SSSS was one of the most important differential diagnoses in the past, but the incidence is currently very low with 0.09 and 0.13 cases per one million inhabitants per year [54].

## Management and Therapy

### Treatment in acute stage

Management in the acute stage involves sequentially evaluating the severity and prognosis of disease, prompt identification and withdrawal of the culprit drug(s), rapidly initiating supportive care in an appropriate setting, and eventual "specific" drug therapy as described in detail below.

#### Rapid evaluation of severity and prognosis

As soon as the diagnosis of SJS or TEN has been established, the severity and prognosis of the disease should be determined so as to define the appropriate medical setting for further management. In order to evaluate prognosis in patients with SJS/TEN, the validated SCORTEN disease severity scoring system can be used (see section devoted to prognosis and Table 2). Patients with a SCORTEN score of 3 or above should be managed in an intensive care unit if possible.

**Table 2:** SCORTEN severity-of-illness score.

SCORTEN Parameter	Individual score	SCORTEN (sum of individual scores)	Predicted mortality (%)
Age > 40 years	Yes = 1, No = 0	0-1	3.2

Malignancy	Yes = 1, No = 0	2	12.1

Tachycardia (> 120/min)	Yes = 1, No = 0	3	35.8

Initial surface of epidermal detachment >10%	Yes = 1, No = 0	4	58.3

Serum urea >10 mmol/l	Yes = 1, No = 0	> 5	90

Serum glucose >14 mmol/l	Yes = 1, No = 0		

Bicarbonate >20 mmol/l	Yes = 1, No = 0		

#### Prompt withdrawal of culprit drug(s)

Prompt withdrawal of causative drugs should be a priority when blisters or erosions appear in the course of a drug eruption. Garcia-Doval et al. have shown that the earlier the causative drug is withdrawn, the better the prognosis, and that patients exposed to causative drugs with long half-lives have an increased risk of dying [62]. In order to identify the culprit drug(s) it is important to consider the chronology of administration of the drug and the reported ability of the drug to induce SJS/TEN. The chronology of administration of a culprit drug, or time between first administration and development of SJS/TEN, is between 1 and 4 weeks in the majority of cases. The reported ability or likelihood of a drug be the cause of SJS/TEN can be found in Pubmed/Medline or other appropriate sources such as the Litt's drug eruption reference manual [63].

#### Supportive Care

SJS/TEN is a life threatening condition and therefore supportive care is an essential part of the therapeutic approach [64]. A multicenter study conducted in the USA [65], and including 15 regional burn centers with 199 admitted patients, showed that survival rate - independent of the severity of disease (APACHE-score and TBSA = Total body surface area) - was significantly higher in patients who were transferred to a burn unit within 7 days after disease-onset compared with patients admitted after 7 days (29.8% vs 51.4% (p < 0.05)). This positive association of early referral and survival has been confirmed in other studies [27,66].

A single center retrospective study on the outcome of patients after admission to a burn center identified sepsis at the time of admission as the most important negative prognostic factor, followed by age, and to a lesser extent the percentage of total body surface area involved. Co-morbidities and the use of steroids may be important on an individual basis, but lose significance in presence of other factors [67].

A critical element of supportive care is the management of fluid and electrolyte requirements. Intravenous fluid should be given to maintain urine output of 50 - 80 mL per hour with 0.5% NaCl supplemented with 20 mEq of KCl. Appropriate early and aggressive replacement therapy is required in case of hyponatraemia, hypokalaemia or hypophosphataemia which quite frequently occur. Wounds should be treated conservatively, without skin debridement which is often performed in burn units, as blistered skin acts as a natural biological dressing which likely favors re-epithelialization. Non-adhesive wound dressings are used where required, and topical sulfa containing medications should be avoided.

#### Drug Therapy

To date, a specific therapy for SJS/TEN that has shown efficacy in controlled clinical trials unfortunately does not exist. Several treatment modalities given in addition to supportive care are reported in the literature and these are discussed below.

- Systemic steroids were the standard treatment until the early 1990's, although no benefit has been proven in controlled trials. In the absence of strong evidence of efficacy, and due to the confusion resulting from the numerous steroid treatment regimens reported (treatment of short versus long duration, various dose regimens), their use has become increasingly disputed. A recent retrospective monocenter study suggests that a short course "pulse" of high dose corticosteroids (dexamethasone) may be of benefit [68]. On the other hand, a recent retrospective case-control study conducted by Schneck et al. in France and Germany concluded that corticosteroids did not show a significant effect on mortality in comparison with supportive care only [69].

- Thalidomide, a medication with known anti-TNFα activity that is immunomodulatory and anti-angiogenetic has been evaluated for the treatment of TEN [70,71]. Unfortunately, in a double-blind, randomised, placebo-controlled study higher mortality was observed in the thalidomide-treated group suggesting that thalidomide is detrimental in TEN.

- High-dose intravenous immunoglobulins. As a consequence of the discovery of the anti-Fas potential of pooled human intravenous immunoglobulins (IVIG) in vitro [44], IVIG have been tested for the treatment of TEN, and their effect reported in different non-controlled studies. To date, numerous case reports and 12 non-controlled clinical studies containing 10 or more patients have analyzed the therapeutic effect of IVIG in TEN (Table 3). All except one study [72], confirm the known excellent tolerability and a low toxic potential of IVIG when used with appropriate precaution in patients with potential risk factors (renal insufficiency, cardiac insufficiency, IgA deficiency, thrombo-embolic risk) [73].

**Table 3:** Summary of studies concerning IVIG for TEN

	Trent	Viard	Prins	Campione	Al-Mutairi	Shortt	Tan	Stella	Rajaratnam	Bachot	Brown	Schneck
	2003	1998	2003	2003	2004	2004	2005	2007	2010	2003	2004	2008
	(80)	(44)	(73)	(76)	(74)	(78)	(79)	(82)	(81)	(72)	(75)	(69)
**Study design**	**Retro**	**Prosp**	**Retro**	**Prosp**	**Prosp**	**Retro**	**Retro**	**Retro**	**Retro**	**Prosp**	**Retro**	**Retro**
	**N/**	**N/**	**N/**	**N/**	**N/**	**N/**	**N/**	**Cont**	**N/**	**N/**	**N/**	**Cont**
	**Cont**	**Cont**	**Cont**	**Cont**	**Cont**	**Cont**	**Cont**		**Cont**	**Cont**	**Cont**	

Patients	24	10	48	10	12	16	12	23	14	34	24	75

Detachm.(%)	44	39	45	49	58	"65"	---	---	"44"	19	49	---

Total dose IVIG (g/kg)	4	3	3	2	2-5	2.8	2	---	3.3	2	1.6	1.9 (0.7-2.3)

Predicted mortality (SCORTEN/APACHE*) in %	33	---	---	35	---	38 *	---	35.8	36	24	28.6	25

Actual mortality in %	4	0	12	10	0	25	8	26	21	32	41.7	34

Taken together, although each study has its potential biases and the 12 studies are not directly comparable, 9 of the 12 studies suggest that there may be a benefit of high-dose IVIG on the mortality associated with TEN [44,69,72,74-82]. Analysis of studies published (Table 3), suggests that total IVIG doses of more than 2 g/kg may be of greater benefit than doses of 2 g/Kg or less. To determine if a dose response relationship exists, Trent et al. analyzed the published literature between 1992 and 2006, selected all studies performed in adults in which the dose of IVIG administered was reported for each patient, excluded cases appearing as duplicates in separate publications where possible, and performed a multivariate logistic regression analysis to evaluate mortality and total IVIG dose after controlling for age and affected body surface area [83]. Although this study has limitations stated by the authors and including publication bias, heterogeneous diagnostic definitions and methods of each study, as well as the exclusion of 2 studies owing to lack of individual IVIG dosing data, logistic regression results showed that, with each 1 g/Kg increase in IVIG dose, there was a 4.2-fold increase in TEN patient survival, which was statistically significant. Patients treated with high doses of IVIG had a significantly lower mortality compared with those treated with lower doses, and notably the mortality was zero percent in the subset of 30 patients treated with more than 3 g/kg total dose of IVIG. Given the favourable side-effect profile of IVIG and the data existing to date, in the authors' opinion early administration of high-dose immunoglobulin (3 g/kg total dose given over 3-4 days) should be considered alongside supportive care for the treatment of toxic epidermal necrolysis, given the absence of other validated specific therapeutic alternatives.

The concomitant administration of corticosteroids or immunosuppressive agents remains controversial. IVIG has also been applied in a few children with SJS/TEN, and two non-controlled studies suggest a possible benefit [84,85].

- Ciclosporin (CsA). CsA, a calcineurin-inhibitor, is an efficient drug in transplantation and autoimmune diseases. Arevalo et al. have performed a study as a case series with two treatment arms: CsA alone versus cyclophosphamide in combination with corticosteroids. Patients treated with CsA had significantly shorter time to complete re-epithelialisation, and fewer patients with multi-organ failure and death were observed [86]. A small case series with three TEN patients treated initially with high-doses of intravenous dexamethasone followed by CsA showed a stop in disease progression within 72 h [87]. Other single case reports also reported a positive effect of the use of CsA in TEN [88,89]. Recently, **Valeyrie-Allanore L **conducted an open, phase II trial to determine the safety and possible benefit of ciclosporin [90]. Twenty-nine patients were included in the trial (10 SJS, 12 SJS-TEN overlap and 7 TEN), and 26 completed the treatment with CsA administered orally (3 mg/kg/d for 10 days) and tapered over a month. The prognostic score predicted 2.75 deaths and none occurred (p = 0.1), suggesting that, although not statistically significant, ciclosporin may be useful for the treatment of TEN.

- TNF antagonists. A new therapeutic approach targeting the proinflammatory cytokine TNFα has been proposed by Hunger et al. They treated one patient with a single dose of the chimeric anti-TNFα antibody (infliximab 5 mg/kg) and reported that disease progression stopped within 24 hours followed by a complete re-epithelialisation within 5 days [91]. Meiss et al. report three cases with an overlap of acute generalized exanthematous pustulosis and TEN and treatment response to infliximab [92]. Administration of the soluble TNFα Receptor Etanercept 25 mg on days 4 and 8 after onset of TEN in a single case resulted in cessation of epidermal detachment within 24 hours but subsequent death of the patient. The published data is currently insufficient to draw a conclusion on the therapeutic potential of TNF antagonists in TEN.

- Plasmapheresis/plasma exchange (PE). PE has also been tried in SJS/TEN, but the current data does not allow a conclusion as to the potential of this approach to be drawn due to the small number of patients treated, the frequent confounding factors including different or combined treatments, and other potential biases [93-95]. Furthermore, a small single retrospective study using PE by Furubacke et al., which compared their case series with two published case series serving as controls, showed no difference in terms of mortality [96].

- Cyclophosphamide (CPP). CPP has been studied in small case series, either in combination with other treatments such as CsA [86], in conjunction with high-dose corticosteroids [97], or alone [98]. Although a beneficial effect of CPP is suggested by the authors of these small trials, larger studies are needed to clarify these preliminary results with a special attention to potential side effects.

### Treatment of sequelae

Due to the often combined involvement of the skin, eyes and mucous membranes (oral, gastrointestinal, pulmonary, genital, as well as urinary), the follow up and treatment of sequelae should be interdisciplinary. Special attention should be given to the prevention of ocular complications. Early referral to an ophthalmologist is mandatory for assessment of the extent of eye involvement and prompt treatment with topical steroids. Visual outcome is reported to be significantly better in patients who receive specific ophtalmological treatment during the first week of disease [23]. Some of the ocular complications have an inflammatory background and have to be treated occasionally with ophthalmic steroids and/or extensive lubrication of the eye [26] in order to prevent progression leading ultimately to the need for corneal transplantation. A small single retrospective study with IVIG showed no significant effect on ocular complications in frequency and severity, but the power of the study was weak [99]. The benefit of local antibiotic treatment (ointments) is not clear. Yip et al. have reported that the use of local antibiotic treatment leads to more late complications, including, for example, dryness of the eyes [24]. Hypopharyngeal stenosis combined with dysphagia and oesophageal strictures are long-term complications which are difficult to treat [100,101] and may require laryngectomy.

### Allergological testing

A detailed drug history is very important when striving to identify the culprit drug in SJS/TEN. In some cases several drugs are possible candidates and allergological testing can be of help in identifying the most likely candidate. In principle, the severity of SJS and TEN does not allow re-challenge and intradermal testing with the culprit drugs due to the feared risk of re-inducing a second episode of SJS/TEN, although two case reports describe intradermal testing without triggering of a second episode of TEN [102,103]. Induction of SJS/TEN has, however, been documented following local eye treatment [104,105].

Patch-testing is an investigational option, but not a routine diagnostic option at the moment. Data from Wolkenstein et al. has shown that low sensitivity is a problem with patch testing in SJS/TEN, as only two of 22 tested patients had a relevant positive patch test [106].

Currently the focus of allergological testing lies more on *ex vivo/in vitro *tests. The lymphocyte transformation test (LTT), that measures the proliferation of T cells to a drug *in vitro *has shown a sensitivity of 60-70% for patients allergic to beta-lactam antibiotics [107]. Unfortunately the sensitivity of the LTT is still very low in SJS/TEN even if performed within one week after onset of the disease [108].

Another recently reported approach looks for up-regulation of CD69 on T-lymphocytes two days after lymphocyte stimulation in vitro as a sign of drug hypersensitivity [109]. Novel *in vitro *methods to help identify culprit drug in SJS/TEN are still needed [110].

## Prognosis

SJS and TEN are severe and life-threatening. The average reported mortality rate of SJS is 1-5%, and of TEN is 25-35%; it can be even higher in elderly patients and those with a large surface area of epidermal detachment [64]. In order to standardize the evaluation of risk and prognosis in patients with SJS/TEN, different scoring systems have been proposed. The SCORTEN is now the most widely used scoring system and evaluates the following parameters: age, malignancy, tachycardia, initial body surface area of epidermal detachment, serum urea, serum glucose, and bicarbonate (Table 2) [111]. Yun et al. reported recently that lactate dehydrogenase (LDH) may be an additional useful parameter in the evaluation of disease severity [112].

More than 50% of patients surviving TEN suffer from long-term sequelae of the disease. These include symblepharon, conjunctival synechiae, entropion, ingrowth of eyelashes, cutaneous scarring, irregular pigmentation, eruptive nevi, and persistent erosions of the mucous membranes, phimosis, vaginal synechiae, nail dystrophy, and diffuse hair loss.

## Abbreviations

AGEP: acute generalized exanthematous pustulosis; CLA: cutaneous leukocyte antigen; CsA: ciclosporine; CTL: cytotoxic T lymphocytes; EM: erythema multiforme; HLA: human leukocyte antigen; IVIG: intravenous immunoglobuline; MHC: major histocompatibility complex; NSAID: non-steroidal anti-inflammatory drug; SJS: Stevens Johnson syndrome; SSSS: staphylococcal scalded skin syndrome; TEN: toxic epidermal necrolysis.

## Competing interests

The authors declare that they have no competing interests.

## Authors' contributions

Both authors made substantial contributions and have given final approval to the version being published.
